# Physical activity in patients with cancer: self-report versus accelerometer assessments

**DOI:** 10.1007/s00520-019-05203-3

**Published:** 2019-12-09

**Authors:** Joeri A.J. Douma, Maaike B. de Beaufort, Caroline S. Kampshoff, Saskia Persoon, Jorine A. Vermaire, Mai J. Chinapaw, Willem van Mechelen, Frans Nollet, Marie José Kersten, Jan H. Smit, Irma M. Verdonck-de Leeuw, Teatske M. Altenburg, Laurien M. Buffart

**Affiliations:** 1 Department of Medical Oncology, Cancer Center Amsterdam, Amsterdam UMC, Vrije Universiteit Amsterdam, De Boelelaan 1117, Amsterdam, The Netherlands; 2 Knowledge Institute of the Dutch Association of Medical Specialists, Mercatorlaan 1200, Utrecht, The Netherlands; 3 Department of Radiation Oncology, Division of Medical Imaging, University Medical Center Utrecht, Utrecht University, Heidelberglaan 100, Utrecht, The Netherlands; 4 Department of Public and Occupational Health, Amsterdam Public Health Research Institute, Amsterdam UMC, Vrije Universiteit Amsterdam, De Boelelaan 1089a, Amsterdam, The Netherlands; 5 School of Human Movement and Nutrition Sciences, Faculty of Health and Behavioural Sciences, University of Queensland, Brisbane, Australia; 6 Division of Exercise Science and Sports Medicine (ESSM), Department of Human Biology, Faculty of Health Sciences, University of Cape Town, Cape Town, South Africa; 7 School of Public Health, Physiotherapy and Population Sciences, University College Dublin, Dublin, Ireland; 8 Department of Rehabilitation, Amsterdam Movement Sciences Research Institute, Amsterdam UMC, University of Amsterdam, Meibergdreef 9, Amsterdam, Netherlands; 9 Department of Hematology, Amsterdam UMC, University of Amsterdam, Cancer Center Amsterdam and LYMMCARE (Lymphoma and Myeloma Center Amsterdam), Meibergdreef 9, Amsterdam, Netherlands; 10 Department of Psychiatry, Amsterdam Public Health Research Institute, Amsterdam UMC, Vrije Universiteit Amsterdam, Oldenaller 1, Amsterdam, The Netherlands; 11 Department of Otolaryngology-Head and Neck Surgery, Cancer Center Amsterdam, Amsterdam UMC, Vrije Universiteit Amsterdam, De Boelelaan 1117, Amsterdam, The Netherlands; 12 Department of Clinical, Neuro- and Developmental Psychology, Section Clinical Psychology, Amsterdam Public Health Research Institute, Amsterdam UMC, Vrije Universiteit Amsterdam, De Boelelaan 1089a, Amsterdam, The Netherlands; 13 Department of Epidemiology and Biostatistics, Amsterdam Public Health Research Institute, Amsterdam UMC, Vrije Universiteit Amsterdam, De Boelelaan 1089a, Amsterdam, The Netherlands

**Keywords:** Agreement, Exercise, Self-reported physical activity, Accelerometer-assessed physical activity, Cancer

## Abstract

**Purpose:**

The level of daily physical activity in patients with cancer is frequently assessed by questionnaires, such as the Physical Activity Scale for the Elderly (PASE). Objective assessments, with for example accelerometers, may be a good alternative. The aim of this study was to investigate the agreement between the PASE questionnaire and accelerometer-assessed physical activity in a large group of patients with different types of cancer.

**Methods:**

Baseline accelerometer and PASE questionnaire data of 403 participants from the REACT (Resistance and Endurance Exercise After Chemotherapy, *n* = 227), the EXIST (Exercise Intervention After Stem-Cell Transplantation, *n* = 74), and NET-QUBIC (NEtherlands QUality of Life And Biomedical Cohort Studies In Cancer, *n* = 102) studies were available for the current analyses. Physical activity was assessed by the PASE questionnaire (total score) and accelerometers (total minutes per day > 100 counts). Linear mixed models regression analysis was used to assess the agreement between the PASE questionnaire and accelerometer-assessed physical activity.

**Results:**

The mean (SD) PASE score was 95.9 (75.1) points and mean (SD) time in physical activity measured with the accelerometer was 256.6 (78.8) min per day. The agreement between the PASE score and the accelerometer data was significant, but poor (standardized regression coefficient (*B*) = 0.36, 95%CI = 0.27; 0.44, *p* < 0.01).

**Conclusion:**

Agreement between the PASE questionnaire and accelerometer-assessed physical activity was poor. The poor agreement indicates that they measure different physical activity constructs and cannot be used interchangeably to assess the level of daily physical activity in patients with cancer.

## Introduction

Regular daily physical activity is associated with a reduced risk of cancer development and a reduction in mortality after the diagnosis of cancer [1–3]. Additionally, physical activity and exercise interventions have significant beneficial effects on the level of fatigue and quality of life in patients with cancer [4–6]. Previous studies have also shown that the magnitude of these beneficial effects is greater in patients with worse baseline values of physical activity [6]. Correct estimation of the levels of physical activity in patients with cancer is of utmost importance to estimate its effect on various health outcomes and to estimate the effectiveness of intervention programs. Furthermore, it might be important to identify physically inactive patients correctly in order to offer these patients an exercise intervention program. Although various measurement methods are available to estimate levels of physical activity of patients with cancer, there is no gold standard for measuring physical activity in daily life on a large scale [7, 8].

Self-reported questionnaires are a frequently used measurement method to assess physical activity, both in clinical care and in a research setting [9, 10]. The “Physical Activity Scale for the Elderly” (PASE) questionnaire is a short (5 to 10 min) 13-item questionnaire, including questions on the frequency and duration of various leisure time, household, and work-related activities[11], and was developed and validated to assess physical activity in people over the age of 65 years [12]. The PASE questionnaire has previously been used in younger patients with cancer [13, 14]. Patients with cancer are often debilitated and therefore have lower levels of physical activity compared with age-matched healthy individuals [15]. Consequently, their physical activity levels may be more comparable to the elderly. It has been used in several research projects in elderly people [13, 16–19] and in patients with various types of cancer [9, 20]. Self-reported questionnaires involve minimal time investment, costs, and participant burden, which favors their use in epidemiological studies and large-scale clinical trials [20, 21]. A disadvantage of self-reported questionnaires is that they are prone to recall bias, response shift, and social desirability bias [22, 23] and are therefore likely to over- or underreport physical activity levels [24]. In contrast, accelerometers provide an objective assessment of daily physical activity based on raw accelerations [25]. Unfortunately, accelerometer assessments are expensive and labour intensive, because they have to be initialized before and read out after use, followed by data processing and analysis [26].

In order to be able to make a balanced decision on the choice of using either one or both instruments, it is important to investigate whether the PASE questionnaire and accelerometers provide similar or different insights into the level of physical activity of patients with cancer under free-living conditions. Two previous studies in relatively small samples of patients with cancer showed poor to fair agreement between the PASE questionnaire and accelerometer-assessed physical activity [20, 27]. The agreement between both measurements might differ significantly across different target populations [8], including cancer type, age [10, 28], gender [10], BMI [29], smoking status [28], and employment status [28] of the participants. Furthermore, for epidemiological purposes, it might be important to accurately distinguish a group of physically active from a group of physically inactive patients [30].

The aim of this study was to investigate the agreement between the level of physical activity assessed by the PASE questionnaire and the accelerometer in a large group of patients with different types of cancer. In addition, differences in agreement across various patient characteristics were examined, as well as the agreement between the instruments to distinguish the most and the least physically active patients.

## Method

### Study design and population

This study used baseline data from three studies in which both PASE and accelerometer data were collected: the Resistance and Endurance exercise After ChemoTherapy (REACT) study [31], the Exercise Intervention after Stem cell Transplantation (EXIST) study [32], and the NETherlands QUality of life and Biomedical cohort studies In head and neck Cancer (NET-QUBIC) study [33]. The REACT study evaluated the effects of a 12-week high-intensity and low-to-moderate intensity supervised resistance and endurance interval exercise intervention on physical fitness, fatigue, and health-related quality of life compared with a waiting list control group in 277 adult patients with cancer (i.e., breast, colon, ovarian, testicular, cervical cancer, and lymphoma) [31]. The EXIST study investigated the effects of an 18-week supervised high-intensity resistance and interval exercise intervention compared with usual care on the same outcomes as the REACT study in 109 patients with multiple myeloma or lymphoma recently treated with high-dose chemotherapy and autologous stem cell transplantation [32]. Patients for both the REACT- and EXIST study were recruited between 2011 and 2014. In the REACT study, baseline assessment took place 4–6 weeks after completion of cancer treatment including ((neo-)adjuvant) chemotherapy and in the EXIST study 6–14 weeks after autologous stem cell transplantation [31, 32]. The NET-QUBIC study is a longitudinal observational cohort study which aims to describe the long-term course of quality of life in 739 newly diagnosed patients with head and neck cancer (HNC) and their informal caregivers and to identify cancer-related, personal, biological, psycho-behavioral, physical, and lifestyle-related and social determinants of quality of life. Baseline assessments took place shortly after the diagnosis of head and neck cancer and before the start of treatment. The total NET-QUBIC assessment protocol involved three components: (1) patient-reported outcome measures; (2) home visit with interviews and tests (including physical fitness), during this home visit, patients were provided with materials to collect data of physical activity (accelerometer) and saliva samples; (3) collection of blood and oral rinse samples. Due to logistic reasons, not all components could always be performed (e.g., short time between diagnosis and start of treatment). Also, patients were allowed not to complete all three components, if this was too much burden. The first data release included baseline data of the first 254 patients who were included between February 2014 and June 2016. All studies were approved by the Medical Ethics Committee of the Amsterdam UMC (VU University Medical Center or Academic Medical Center) and the local ethical boards of all participating hospitals. All patients provided written informed consent prior to participating in the respective studies.

### Outcome measurements

#### The PASE questionnaire

The PASE questionnaire assesses the duration and frequency of physical activities that have been undertaken in the past 7 days [34]. The PASE questionnaire contains 13 questions on leisure, household, and paid or unpaid work-related physical activities. First, patients are asked to estimate the frequency of a particular type of physical activity per week, for which they can choose between never, seldom (1–2 days per week), sometimes (3–4 days per week), or often (5–7 days per week). Second, patients are asked to estimate the duration of that particular type of activity, for which patients could choose between < 1 h, between 1 and 2 h, between 2 and 4 h, and > 4 h for the leisure and household activities. For the work activity, different categories for duration are used: < 1 h, between 1 and 4 h, 5 and 8 h, and > 8 h. For each different types of physical activity, there are published weightings available for the respective PASE scores, based on estimated metabolic equivalent of task (METs) and accelerometer-assessed physical activity [11]. Each physical activity–specific weighting is multiplied by a factor based on the frequency and the duration of this activity, to calculate the activity-specific subscore [35]. Finally, the total score of the PASE questionnaire was calculated by totaling the subscores of all activities [35]. Only fully completed PASE questionnaires were used for this analysis.

#### The accelerometer

Patients were instructed to wear an accelerometer (ActiGraph wGT3X, ActiGraph LLC, Pensacola, FL, USA) on the hip for seven (REACT and NET-QUBIC study) or five (EXIST study) consecutive days to measure physical activity. This accelerometer measures raw accelerations (i.e., epochs; the rate of change of the velocity) in three axes [7]. Vertical accelerations were converted into counts per minute, with several data reduction steps [36]. Non-wear time was defined as ≥ 60 consecutive minutes of consecutive zeros and a valid day was defined as ≥ 10 h/day of wear time [37, 38]. To be included in the analyses, a patient needed to have at least five valid days, including one weekend day. Total time spent in physical activity was defined as the total of all the time periods with ≥ 100 counts per minute [39] and was calculated for each valid wear day. The sum of all physically active minutes during all valid wear days was divided by the number of valid wear days, in order to calculate the mean number of minutes of physical activity per day. Furthermore, the accelerometer-assessed physical activity was also expressed in counts per minute, because in this way, the intensity of physical activity is also taken into account. Counts per minute were calculated by summing up the total counts and divided by the total wear time during all valid measurement days.

#### Potential effect modifiers

Age, gender, and cancer type were retrieved from the patients’ medical records. Body weight and height were measured and body mass index (BMI) was calculated based on these measurements (body weight/height^2^, kg/m^2^). The overall comorbidity score in the NET-QUBIC study was rated as none, mild, moderate, or severe with the use of the Adult Comorbidity Evaluation-27 (ACE-27) [40]. The comorbidity level of participants in the EXIST study was assessed by a sports physician and by a custom-made patient-reported questionnaire and was retrieved from the patients’ medical records. In the REACT study, the number of comorbidities was retrieved from the medical records and calculated as the sum of each of the following conditions: heart disease, lung disease, diseases of the digestive system, diseases of the nervous system, endocrine disease, mental disorder, rheumatism or arthritis, chronic pain, and other conditions. Subsequently, for all the studies, the number of comorbidities was dichotomized into any versus none. The highest level of education, alcohol consumption, and smoking behavior were assessed through study-specific questions. The highest level of education was dichotomized into low/moderate (primary vocational education to senior general secondary education) versus high education level (higher general secondary education, higher professional education, and university). For smoking behavior and alcohol consumption, all patients who smoked or consumed alcohol at the time of the study were defined as smokers and consumers of alcohol, respectively. Information about alcohol consumption was only available in the NET-QUBIC study.

### Statistical analysis

Descriptive statistics (mean, standard deviation (SD), or numbers and percentages) were generated for demographic, clinical, lifestyle-related factors, total PASE score, and accelerometer-assessed physical activity. The agreement between the PASE questionnaire (total score) and accelerometers (minutes of physical activity per day) was analyzed with a linear mixed models regression analysis, with the total score of the PASE questionnaire as independent variable and accelerometer-assessed physical activity as dependent variable. A random intercept on study level was added to take clustering of patients from different studies into account. Standardized regression coefficients (*B*) and the corresponding 95% confidence intervals (CI) were reported. To check for the differences in agreement across different patient characteristics (age, gender, BMI, education, comorbidity, alcohol, and smoking), we added these variables and their interaction terms with the PASE score into the regression model separately for each characteristic. Significant effect modification was defined as a *p* value of < 0.05 of the interaction term in the multilevel mixed models regression analysis [41]. If significant effect modification was found, stratified analyses were performed. A standardized regression coefficient of 0.50 or higher was considered as fair agreement and a coefficient of 0.70 or higher was considered as good agreement [42]. A scatter plot was used to visualize the agreement between the PASE questionnaire and accelerometer-assessed physical activity. We performed a sensitivity analysis in which accelerometer-based physical activity was expressed in counts per minute.

To investigate the agreement between both measurement methods for the identification of the most and the least physically active patients, the highest active quartile of patients based on the accelerometer was compared with the highest active quartile of patients based on the PASE questionnaire. Crosstabs were generated to present the proportion of patients that were both in the highest quartile of the PASE score as well as in the highest quartile of the accelerometer-assessed physical activity. This process was repeated for the lowest quartiles of physical activity. All statistical analyses were performed with SPSS version 22 (IBM Corporation, Armonk, NY, USA).

## Results

**Fig. 1:**
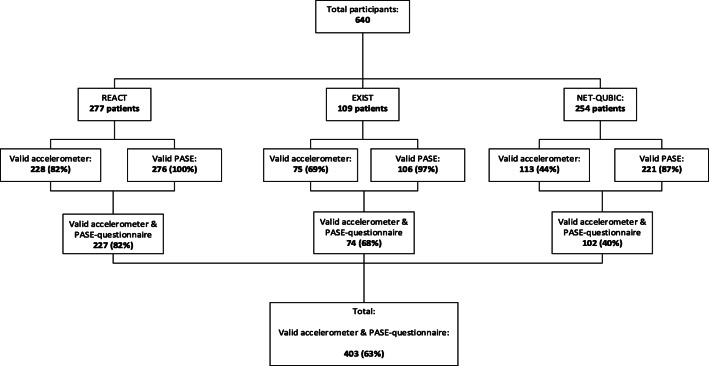
Flowchart of patients with valid measurements

In total, 640 patients participated in the REACT (*n* = 277), EXIST (*n* = 109), and NET-QUBIC studies (*n* = 254). In 38 patients in the NET-QUBIC study, a home visit was not performed and thus had no measurements of physical activity and another 53 patients did not have enough time between diagnosis and start of treatment to measure sufficient valid wear days. Other reasons for missing data for the total group of patients were insufficient valid accelerometer wear days by participants (*n* = 122), technical problems with the accelerometer (*n* = 11), and incomplete or missing PASE questionnaire (*n* = 37) (Fig. 1). In total, 403 patients were included in the current analyses because they had both valid accelerometer data and a complete PASE questionnaire (Fig. 1). The mean (SD) age of these 403 patients was 56 (11) years, 58% of the patients were women, and breast cancer was the most common cancer type (36%), followed by head and neck cancer (25%) and lymphoma (13%) (Table 1). The mean (SD) score of the PASE questionnaire was 95.9 (75.1) and the mean (SD) accelerometer-assessed physical activity was 256.6 (78.8) minutes of physical activity per day (Table 1).

**Table 1:** Baseline characteristics, PASE-score, and accelerometer-assessed physical activity

	Total population N=403	REACT study N=227	EXIST study N=74	NET-QUBIC study n=102
Age, mean (SD) years	56.2 (11)	54.1 (11)	53.2 (9)	63.2 (9)
Gender, n (%) male	169 (42)	46 (20)	48 (65)	75 (74)
Cancer type, n (%)				
Breast cancer	144 (36)	144 (63)		
Head and neck cancer	102 (25)			102 (100)
Lymphoma	54 (13)	20 (9)	34 (46)	
Multiple myeloma	40 (10)		40 (54)	
Colon cancer	45 (11)	45 (20)		
Ovarian cancer	9 (2)	9 (4)		
Testicular cancer	5 (1)	5 (2)		
Cervical cancer	4 (1)	4 (2)		
BMI, mean (SD) kg/m^2^	26.5 (4.5)	27.0 (4.4)	25.6 (4.6)	25.9 (4.6)
BMI category, n (%)				
Underweight (BMI <18.5)	8 (2)	1 (0)	2 (3)	5 (5)
Normal weight (BMI 18.5-25)	161 (40)	88 (39)	35 (47)	38 (38)
Overweight (BMI 25-30)	156 (39)	89 (39)	24 (32)	42 (43)
Obesity (BMI > 30)	76 (19)	49 (22)	13 (18)	14 (14)
Educational level, n (%)				
Low/intermediate	259 (64)	139 (61)	48 (65)	72 (71)
High	140 (35)	86 (38)	26 (35)	28 (28)
Unknown	4 (1)	2 (1)		2 (2)
Comorbidity, n (%)				
No	303 (75)	206 (91)	58 (78)	39 (38)
Yes	97 (24)	21 (9)	16 (22)	60 (59)
Unknown	3 (1)	0 (0)	0 (0)	3 (3)
Alcohol consumption, n (%)				
No	27 (7)			27 (27)
Yes	75 (19)			75 (74)
Unknown	301 (75)	227 (100)	74 (100)	0 (0)
Smoking, n (%)				
No	352 (87)	12 (93)	67 (91)	73 (72)
Yes	46 (11)	12 (5)	7 (10)	27 (27)
Unknown	5 (1)	3 (1)	0 (0)	2 (2)
PASE-score, mean (SD)	95.9 (75.1)	100.6 (73.5)	87.5 (80.6)	91.3 (74.2)
Accelerometer assessed physical activity				
mean (SD) minutes in physical activity per day	256.6 (78.8)	280.3 (71.5)	216.3 (64.4)	233.2 (85.3)
mean (SD) activity counts per minute	230.8 (105.8)	256.0 (98.5)	192.9 (88.9)	202.3 (117.8)

*SD*, standard deviation; *n*, number of patients; *BMI*, body mass index; *PASE*, Physical Activity Scale for the Elderly; *REACT*, the Resistance and Endurance exercise After ChemoTherapy study; *EXIST*, the Exercise Intervention after Stem cell Transplantation study

**Fig. 2:**
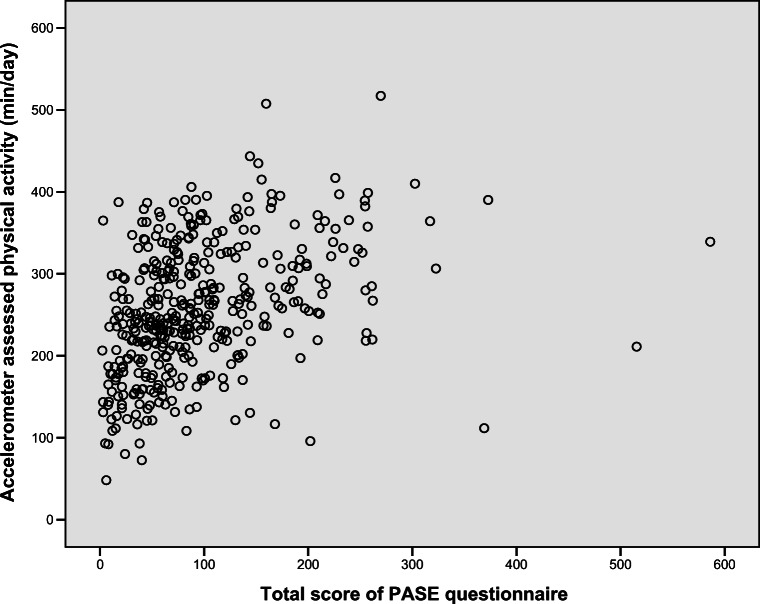
Scatter plot between the total score of the PASE questionnaire and accelerometer-assessed physical activity (min/day)

The agreement between the PASE questionnaire and accelerometer-assessed physical activity expressed in min/day was significant but poor (*B* = 0.36, 95%CI = 0.27; 0.44, *p* < 0.01) (Table 2 and Fig. 2). The agreement between the PASE score and accelerometer output expressed in counts per minute was also poor (*B* = 0.26, 95%CI = 0.17; 0.35, *p* < 0.01). No significant differences in agreement were found between the PASE questionnaire and the accelerometer-assessed physical activity across subgroups of patients (Table 2).

**Table 2:** Agreement between PASE questionnaire and accelerometer-assessed physical activity and potential effect modifiers

Agreement between PASE questionnaire and accelerometer-assessed physical activity	Standardized coefficient (95%CI)	p-value
Total population	0.36 (0.27;0.44)	<0.01
REACT study	0.42 (0.30;0.54)	<0.01
EXIST study	0.36 (0.14;0.58)	<0.01
NET-QUBIC study	0.32 (0.13;0.51)	<0.01
Effect modifiers	Standardized coefficient (95%CI)	P_interaction_
Age	0.00 (-0.00;0.01)	0.32
Age, < 65 vs ≥ 65 years	0.20 (-0.04;0.44)	0.10
Gender, men vs women	-0.06 (-0.25;0.12)	0.50
BMI		
Normal weight	REF	REF
Overweight	0.13 (-0.19;0.22)	0.90
Obesity	0.10 (-0.12;0.32)	0.38
Educational level, low/intermediate vs high	-0.00 (-0.19;0.18)	0.97
Comorbidity, yes vs no	0.01 (-0.19;0.21)	0.92
Alcohol consumption, yes vs no	0.06 (-0.34;0.47)	0.75
Smoking, yes vs no	-0.10 (-0.35;0.15)	0.43

*PASE*, Physical Activity Scale for the Elderly; *NET-QUBIC*, NETherlands QUality of life and Biomedical cohort studies In head and neck Cancer; *REACT*, the Resistance and Endurance exercise After ChemoTherapy study; *EXIST*, the Exercise Intervention after Stem cell Transplantation study; *REF*, reference category

Of the 101 patients in the lowest quartile of physical activity based on accelerometers, 51 were also in the lowest quartile based on the PASE score, indicating an agreement of 50% (Table 3). Forty-four of the 100 patients in the highest quartile of physical activity based on accelerometers were also in the highest quartile based on the PASE-score, indicating an agreement of 44% (Table 3).

**Table 3:** Distribution of patients across lowest and highest quartiles of PASE questionnaire and accelerometer-assessed physical activity

	In lowest quartile *Accelerometer*	Not in lowest quartile *Accelerometer*	Agreement, %
In lowest quartile *PASE-score*	51	49	50
Not in lowest quartile *PASE-score*	50	253	
In highest quartile *PASE-score*	44	56	44
Not in highest quartile *PASE-score*	56	247	

*PASE*, Physical Activity Scale for the Elderly

## Discussion

This study investigated the agreement between the PASE questionnaire and accelerometer-assessed physical activity in a large group of patients with cancer. Results showed that the agreement was poor in all subgroups of patients. The results are in line with the poor agreement reported in an earlier study in 48 patients with different types of cancer [27] and the poor-to-fair agreement in patients with lung cancer [20]. Furthermore, in a recent review on the agreement between accelerometer-assessed physical activity and various physical activity questionnaires among healthy persons and patients with various chronic diseases, only 11 of the 57 included studies reported an agreement (Pearson correlation coefficient) between questionnaires and accelerometers of ≥ 0.50, and for most studies only in specific subgroups [10].

The poor agreement between the PASE questionnaire and accelerometer-assessed physical activity might reflect the complexity of obtaining an integral estimation of all aspects of a highly varying behavior such as physical activity [43]. Low-intensity physical activities, which are more typical for patients with cancer compared with those for the general population [44], might be more often overestimated with self-report [43]. The poor agreement between the PASE score and accelerometer-assessed physical activity might also be caused by social desirability and recall bias in the PASE questionnaire [27]. On the other hand, accelerometers also have disadvantages, which might have influenced the agreement with the PASE questionnaire [7]. For example, the use of hip-placed accelerometers may have underestimated upper body movements and activities such as cycling, swimming, and resistance exercises [27]. In addition to the poor agreement for the absolute levels of physical activity, results also showed poor agreement in distinguishing physically active from physically inactive patients. This finding implies that both measurement methods do not measure the same construct and that they cannot be used interchangeably.

A strength of this study is the sample size of more than 400 patients with various types of cancer and treatment regimens. A limitation of the study might be that most of the patients participated in exercise intervention studies or were recently diagnosed with cancer and were all treated with curative intent, which may have resulted in an under- or overestimation of the levels of physical activity. However, considering the variation in physical activity levels, it is unlikely that this has affected the agreement between the PASE questionnaire and accelerometer-assessed physical activity. Another limitation may be that we used the PASE questionnaire that is originally developed for elderly above 65 years in a group of patients with cancer with a mean age of 56 years. However, we found no significant differences in agreement between patients below and above 65 years of age.

The low agreement between the PASE questionnaire and accelerometer output might indicate that these instruments measure different aspects of the construct physical activity. Therefore, it may be recommended to use both measures in research and in clinical practice, as they may produce complementary information. Physical activity questionnaires might especially be suitable to investigate the mode, type, and frequency of moderate-to-vigorous activities that patients perform, but are generally less accurate to estimate light-intensity physical activities [7]. On the contrary, accelerometers provide an objective estimate of the duration and intensity of physical activity, have no risk of social desirability, response shift and recall bias, but provide limited information about the type of physical activities performed [7]. For optimal use in clinical practice, it is important to reduce the labor intensity and costs of accelerometers or to search for alternatives for objective physical activity assessments [45]. Widely available smartphones, pedometers, or fitness trackers may provide such alternatives, and have shown to be feasible, and produce valid and reliable step counts in patients with cancer, when compared with accelerometers [46, 47].

In conclusion, the results of this study showed a poor agreement between the PASE questionnaire and accelerometer-assessed physical activity in patients with cancer. This indicates that they can better be used simultaneously than interchangeably to assess daily physical activity in patients with cancer, both for research and clinical purposes.
